# 1α,25(OH)_2_ Vitamin D_3_ Signaling in Adipose Tissue: Bridging Classical and Non-Classical Pathways in Metabolic Regulation Complexity

**DOI:** 10.3390/nu18122026

**Published:** 2026-06-22

**Authors:** Alice Lima Rosa Mendes, Paola Miranda Sulis, Murilo Ferenz, Bruna Antunes Zaniboni, Marcela Aragón, Guilherme Brasil Pintarelli, Daniela Ota Hisayasu Suzuki, Carine Royer, Fátima Regina Mena Barreto Silva

**Affiliations:** 1 Instituto de Bioeletricidade Celular (IBIOCEL): Ciência & Saúde, Departamento de Bioquímica, Centro de Ciências Biológicas, Universidade Federal de Santa Catarina, Rua João Pio Duarte Silva 241, Sala G301, Córrego Grande, Florianópolis 88037-000, Brazil; alice.rosa@posgrad.ufsc.br (A.L.R.M.); paolamsulis@gmail.com (P.M.S.); ferenzmurilo@gmail.com (M.F.); brunaantunesz@outlook.com (B.A.Z.); dmaragonn@unal.edu.com (M.A.); guilherme.pintarelli@ufsc.br (G.B.P.); daniela.suzuki@ufsc.br (D.O.H.S.); cariroyer@unb.br (C.R.); 2 Departamento de Farmacia, Universidad Nacional de Colombia, Av. Carrera 30 45-03 Edif. 450, Bogotá 111321, Colombia; 3 Departamento de Engenharia de Controle, Automação e Computação, Universidade Federal de Santa Catarina, Blumenau 89065-100, Brazil; 4 Instituto de Engenharia Biomédica, Engenharia Elétrica, Centro Tecnológico, Universidade Federal de Santa Catarina, Florianópolis 88037-000, Brazil; 5 Laboratório de Farmacologia Molecular, Universidade de Brasília, Brasília 70900-910, Brazil; 6 Faculdade de Ciências e Tecnologias em Saúde, Universidade de Brasília, Brasília 70900-910, Brazil

**Keywords:** vitamin D, adipocyte, signaling, vitamin D receptor, AMP-activated protein kinase, sirtuin 1, microRNA

## Abstract

Background: Adipose tissue is increasingly recognized as a highly dynamic endocrine and immunometabolic organ with marked functional heterogeneity. It serves as a reservoir for the active form of vitamin D_3_, 1α,25-dihydroxyvitamin D_3_ or calcitriol (1α,25-D_3_), since it expresses enzymes responsible for its activation and inactivation and contains the vitamin D receptor (VDR). Through both classical and non-classical mechanisms, calcitriol modulates adipocyte proliferation and differentiation, protein expression and energy metabolism. This review aims to explore the signal transduction mechanisms of calcitriol in adipocytes, detailing the classical pathways mediated by the nuclear VDR (VDRn), as well as non-classical pathways involving membrane-associated VDR (VDRm), microRNAs, AMP-activated protein kinase (AMPK), and sirtuin 1 (SIRT1). Methods: A literature search was conducted using PubMed, ScienceDirect, and MDPI-indexed journals, prioritizing studies published within the last 10 years to ensure the inclusion of up-to-date evidence. Results: This review summarizes current knowledge on both classical and non-classical signaling pathways that are activated by calcitriol and highlights key molecular targets with potential relevance for drug development and therapeutic intervention. Through VDRn, calcitriol regulates the expression of proteins involved in inflammation and energy metabolism. Additionally, it modulates cellular processes such as energy production and secretion via the AMPK/SIRT1 axis and microRNA-mediated pathways, contributing to mitochondrial function and metabolic homeostasis. Conclusions: Calcitriol plays a central role in adipocyte biology by integrating multiple signaling pathways that regulate metabolic and inflammatory responses. These mechanisms highlight its potential as a therapeutic target and biomarker in metabolic diseases. Moreover, microRNAs emerge as critical posttranscriptional regulators in these processes, reinforcing their relevance as both biomarkers and targets for future interventions.

## 1. Introduction

### 1.1. Adipose Tissue Beyond Energy Storage and Immunometabolism Function: Where and How Does 1α,25(OH)_2_ Vitamin D_3_ Fit in?

White adipose tissue (WAT), known as the major reservoir of triglycerides, can remove fatty acids to provide energy to the body by dynamically balancing uptake, storage and delivery of fatty acids. In addition, WAT secretes hormones such as adipokines (leptin, adiponectin, resistin and visfatin) and microRNAs (miRNAs), derived from adipose tissue and identified as novel paracrine, autocrine and endocrine modulators that maintain signaling between different tissues. Moreover, although not the main focus of this review, it is worth mentioning that classical proinflammatory mediators (interleukin-6, interleukin-1β and tumor necrosis factor-α) are also secreted from adipose tissue [1,2,3,4], highlighting its role as an endocrine organ that is associated with immunometabolism function, and reflecting an understanding of the functional evolution of adipose tissue. Furthermore, an important point to address is that the prevalence of obesity and persistent high-fat diet consumption trigger inflammatory processes, inducing insulin resistance, which may lead to type 2 diabetes mellitus. The summatory of these factors characterizes a metabolic disorder with severe consequences to the body, such as impaired glucose tolerance, dyslipidemia and fatty liver, and leading to insulin resistance [5,6].

The main WAT compartments (subcutaneous adipose tissue (SAT) and visceral adipose tissue (VAT)) exhibit marked differences in embryonic origin, vascularization, cellular composition, and secretory profile [7,8]. SAT, predominantly located in peripheral regions, displays a greater capacity for expansion through adipocyte hyperplasia, a lower degree of basal inflammation, and a less proinflammatory secretory profile, and is generally associated with a metabolically protective phenotype [9]. In contrast, VAT, distributed within the abdominal cavity and closely associated with visceral organs, is characterized by higher lipolytic activity, increased infiltration of immune cells, particularly proinflammatory macrophages, and enhanced secretion of cytokines such as TNF-α, IL-6, and MCP-1, being strongly associated with insulin resistance and increased cardiometabolic risk [10,11]. Moreover, the relative proportion of VAT to SAT, independent of body mass index, has emerged as a critical determinant of individual metabolic risk [12]. This structural and functional heterogeneity directly influences local vitamin D_3_ metabolism, as different adipose depots exhibit distinct capacities for storage, activation, and responsiveness to calcitriol (1α,25-D_3_) [13,14].

The reduced responsiveness of VAT to calcitriol may be attributed to a complex interplay between inflammatory factors, alterations in VDR signaling, and changes in the cellular microenvironment. VAT exhibits a state of chronic low-grade inflammation, characterized by increased macrophage infiltration and secretion of proinflammatory cytokines, which may directly interfere with VDR signaling and reduce the metabolic effects of calcitriol [11,15]. Activation of inflammatory pathways such as NF-κB may inhibit VDR transcriptional activity and impair calcitriol-dependent gene regulation [13]. Furthermore, alterations in VDR expression and functionality have been described in the context of obesity and inflammation. Reduced VDR expression or post-translational modifications affecting its activity may limit the responsiveness to calcitriol in VAT [14,16]. Another critical component is the VAT microenvironment, characterized by hypoxia, extracellular matrix remodeling, and increased oxidative stress. Local hypoxia, mediated by HIF-1α activation, is associated with inflammation, mitochondrial dysfunction, insulin resistance, and may also interfere with calcitriol signaling [17,18]. In parallel, alterations in tissue architecture may impair molecular diffusion and local calcitriol bioavailability [19]. Together, these factors create an adverse cellular environment in which the action of calcitriol is attenuated, contributing to the maintenance of metabolic dysfunction, which is characteristic of VAT.

### 1.2. Adipose Tissue Heterogeneity and Implications for the Action of 1α,25(OH)_2_ Vitamin D_3_

Far from representing a uniform lipid storage compartment, adipose tissue comprises distinct anatomical depots with diverse metabolic, inflammatory, and endocrine properties. These depots respond differentially not only to hormonal and nutritional stimuli, such as calcitriol [14,16], but also to recently identified first messengers, including extracellular vesicles rich in microRNAs [20].

In addition to acting as a reservoir, predominantly storing cholecalciferol (vitamin D_3_), adipose tissue expresses key enzymes involved in the activation and inactivation of this hormone, as well as the calcitriol receptor, enabling the autocrine and paracrine actions of calcitriol [1α,25-D_3_] within the adipose microenvironment [13,14]. This metabolic capacity positions adipose tissue as an important modulator of systemic calcitriol bioavailability and as a direct target of its biological actions, including the regulation of adipogenesis, lipid metabolism, mitochondrial function, and inflammatory responses [16,21,22]. Adipose tissue heterogeneity poses significant challenges for the interpretation of experimental and clinical studies. Widely used cellular models, such as 3T3-L1-derived adipocytes, do not fully recapitulate the differences between human adipose depots, particularly regarding the inflammatory microenvironment and VDR regulation [8]. Moreover, the response to calcitriol may be modulated by the metabolic state of cells, including insulin resistance, inflammation, and oxidative stress. Thus, adipose tissue distribution should be considered a critical factor when investigating the metabolic effects of calcitriol and developing targeted therapeutic strategies for obesity and type 2 diabetes [16,22]. Collectively, these findings suggest that adipose tissue should not be treated as a homogeneous compartment. The interplay between inflammation, VDR signaling, and the cellular microenvironment determines the differential response to calcitriol among adipose depots, highlighting the importance of stratified approaches for investigating the biology of calcitriol in the metabolic context.

In vitro and animal studies suggest that calcitriol modulates lipid metabolism through VDR-dependent mechanisms. VDR activation influences the expression of genes involved in adipogenesis, such as *PPARG* and *CEBPA*, and modulates enzymes related to lipogenesis and lipolysis [13,21]. With regard to lipogenesis, evidence indicates that calcitriol may exert bidirectional effects depending on the stage of cellular differentiation and the metabolic context. In early adipogenesis, calcitriol may inhibit adipocyte differentiation, whereas in mature adipocytes it may promote lipid accumulation under certain conditions [16,21]. Studies on lipogenesis demonstrate that calcitriol may modulate enzymes such as hormone-sensitive lipase (HSL) and adipose triglyceride lipase (ATGL), although findings are inconsistent and highly dependent on the experimental model used [13].

Additionally, calcitriol has been associated with the regulation of fatty acid oxidation, possibly through modulation of pathways related to mitochondrial function and energy metabolism, including AMPK activation and increased mitochondrial biogenesis [22,23]. These effects suggest a potential role for calcitriol in improving metabolic efficiency and reducing ectopic lipid accumulation. Conversely, in visceral adipose tissue, findings are more heterogeneous and often indicate reduced responsiveness to calcitriol, suggesting the existence of a functional resistance to this molecule [18]. Additionally, the accumulation of vit D_3_ (cholecalciferol) in adipose tissue, particularly in VAT in individuals with obesity, may reduce its systemic bioavailability, contributing to decreased circulating levels of 25-hydroxycholecalciferol and to a functional state of deficiency despite elevated body stores [19,24].

### 1.3. Distinct Signaling Pathways for 1α,25(OH)_2_ Vitamin D_3_: Classical and Non-Classical Signaling Pathways

The classical understanding of the action of calcitriol has been largely centered on the activation of the vitamin D_3_ receptor (VDR), a nuclear transcription factor that regulates genes associated with calcium homeostasis, cellular differentiation, and immunomodulation. Furthermore, for ionic homeostasis, centered on calcium and phosphate, calcitriol acts at plasma membrane vitamin D receptor (VDRm) [25,26,27]. However, more recent evidence has demonstrated that calcitriol exerts effects that extend beyond this canonical pathway, involving non-classical mechanisms directly related to cellular energy metabolism. In this context, calcitriol is increasingly recognized as a systemic metabolic modulator, capable of integrating energy signals and influencing cellular bioenergetics, particularly in tissues such as skeletal muscle, adipose tissue, and liver [28,29,30,31].

#### 1.3.1. 1α,25(OH)_2_ Vitamin D_3_-Driven Epigenetic Modulation in Adipose Tissue: Unveiling microRNA-Mediated Mechanisms

Mechanisms related to increased adiposity and associated metabolic disorders have focused on gene regulation and the secretion of compounds such as adipokines, including non-peptide effectors such as fatty acid metabolites [32,33]. Furthermore, both long and short non-coding RNAs (ncRNAs) have been found, in recent decades, to control the formation and function of tissues and organs. It is worth highlighting that long ncRNAs (lncRNAs) play an important role in the control of adipogenesis and obesity [34].

To date, small ncRNAs have been extensively studied, with the best-characterized class being microRNAs (miRNAs), which were discovered in 1993 by Lee, Feinbaum, and Ambros [35]. Their impact on diseases is recognized because they can serve as candidates for use as drugs and/or therapeutic targets, as well as disease biomarkers [36,37,38,39,40].

MiRNAs are small, endogenous, non-coding, single-stranded RNAs, which are approximately 22 nucleotides long. They regulate gene expression by binding to their complementary sites within the 3′-untranslated regions (3′ UTRs) of target mRNAs [41], resulting in the repression of mRNA translation or transcript degradation. The degree of base-pairing complementarity between the miRNA and the target determines the result of the target transcription. In fact, miRNAs that bind to the 3′ UTR region of mRNA with imperfect complementarity block the translation of target protein, while miRNAs that bind to mRNA with perfect complementarity induce cleavage of the target mRNA [31,41,42]. Each miRNA can have hundreds of mRNA targets, just as a single mRNA can be regulated by several distinct miRNAs, increasing the complexity of protein expression [42]. In fact, they act selectively on numerous mRNA molecules to repress their expression, thereby modulating a wide range of signaling pathways, including the regulation of adipocyte differentiation, lipid metabolism, and obesity [43,44,45,46,47]. Furthermore, miRNAs have emerged as an important class of post-transcriptional regulators of metabolism in various cell types, including muscle cells, adipocytes, and β-cells [48]. Many studies have reported the association of miRNAs with numerous metabolic dysfunctions, particularly in adipose tissue, by controlling adipogenesis, insulin signaling, inflammation, and oxidative stress [43,49,50,51]. Some of these miRNAs are secreted into vesicles and actively participate in intercellular communication [20,52,53,54]. The discovery of circulating miRNAs in exosomes highlights their importance both as endocrine signaling molecules and as potential disease biomarkers.

Adipocyte differentiation occurs through three main transcriptional events: (1) transcriptional induction of the CCAAT-binding protein/enhancer β (*CEBPB*) and CCAAT binding protein/enhancer δ(CEBPD) genes; (2) activation of PPARγ and C/EBPα, also considered master regulators of adipogenesis; and (3) upregulation of adipocyte-specific genes, such as *SREBF1*, *FASN*, *FABP4*, hormone-sensitive lipase *LIPE*, lipoprotein lipase (*LPL*), stearoyl-CoA desaturase (*SCD*), acetyl-CoA carboxylase (ACACA) [55,56,57]. This adipogenic differentiation can be regulated in many ways, including by miRNAs, which play important roles in this process [46,47,58].

Several anti-adipogenic miRNAs have been identified in mice, humans, cattle and pigs, such as let-7, miR-24, miR-27a/b, miR-31, miR-130, miR-135a, miR-137, miR-138, miR-215, miR-302a, miR-344, miR-375, and miR-448 [59,60]. These miRNAs negatively affect adipogenesis by repressing genes involved in key transcriptional activities and signaling pathways [31,51,58,59,61].

Thus, it has been reported that miR-27a and miR-27b function as negative regulators of adipocyte differentiation by inhibiting PPARγ [59,62]. Natural compounds, such as persimmon tannin [63], and even flavonoid derivatives [64] could inhibit the differentiation of 3T3-L1 cells and positively regulate miR-27a/b expression during the differentiation process [63,64]. These studies suggest that the miR-27 family could be a useful antiadipogenic target for the treatment of obesity.

In 2012, Yunxue Guo et al. [61] first reported on the inhibitory role of miR-145 in the differentiation of porcine pre-adipocytes, acting on insulin receptor substrate 1 (IRS1). It was demonstrated that miR-145 undergoes downregulation during human adipocytes differentiation [61]. In 2016, the inhibitory effect of miR-145 on adipogenesis was again confirmed in 3T3-L1 cells [65]. In 2020, miR-145 was found to inhibit adipogenesis in bovine preadipocytes by reducing the activity of the PI3K/Akt and MAPK signaling pathways, and decreasing the expression of PPARγ, CCAAT/enhancer-binding protein alpha (C/EBPα), and fatty acid-binding protein 4 (*FABP4*). This effect was reversed by insulin, a potent inducer of adipogenesis [31]. The role of miR-145 in lipolysis has been reported inconsistently across different studies [66,67]. In primary adipocytes isolated from mice epididymal fat depots, miR-145 acts as an important negative regulator of lipolysis [67]. On the other hand, in human subcutaneous WAT, miR-145 upregulates adipocyte lipolysis [66]. These conflicting results may be due to interspecies differences or varying experimental protocols.

There are also several pro-adipogenic microRNAs. The first miRNA associated with adipogenesis to be described was miR-143, which plays a positive role in adipocyte differentiation by targeting extracellular signal-regulated kinase 5 (ERK5) [68]. Subsequently, a considerable number of miRNAs that positively regulate adipose differentiation have been reported [44].

The miR-26 family, composed of miR-26a and miR-26b, promotes adipogenesis by targeting A disintegrin metallopeptidase domain 17 (ADAM17), which cleaves Pref-1, a marker of adipocyte precursors. This molecule is downregulated during adipocyte differentiation [44]. MiR-30c is also induced during adipocyte differentiation from human multipotent adipose-derived stem cells (hMADS) [69]. Since genomic studies have revealed that a single miRNA can directly regulate hundreds of target mRNAs [70,71], for any biological process under investigation, it is therefore obvious that (1) identifying the targets that mediate the effects of miRNAs is a challenge; and that (2) the miRNA likely mediates its effects through more than one single target. The direct interaction of miR-30c with PAI-1 and ALK2 via unique miRNA binding sites in their 3′ UTR region serves as a possible link between two distinct pathways, demonstrating that miRNAs can connect and coordinate large regulatory networks [69].

MiRNAs can also promote adipogenesis by inhibiting anti-adipogenic Wnt signaling. MiR-148a is a CREB-dependent adipogenesis-specific miRNA in human adipose-derivate mesenchymal stem cells, which mediates its effect by modulating adipogenesis-inhibiting Wnt signaling. It is important to note that miR-148a, as a biomarker of obesity, in humans and murine models, represents a CREB-regulated miRNA that acts to repress Wnt1, thereby promoting adipocyte differentiation [72]. MiR-183 attenuates the binding of the Low-Density Lipoprotein Receptor-Related Protein 6 co-receptor to Wnt proteins, leading to the inactivation of the canonical Wnt/β-catenin signaling pathway, decreased accumulation of nuclear β-catenin, and the inhibition of c-myc expression, thereby promoting adipogenesis of 3T3-L1 cells [73].

Adipogenesis involves cell proliferation and differentiation, both of which can be regulated by miRNA [46,74,75]. During the clonal expansion phase of 3T3-L1 pre-adipocyte differentiation, miR-17-92, a miRNA cluster that promotes cell proliferation in various types of cancer [76,77], exhibits significant upregulation. Stable transfection of 3T3-L1 cells with miR-17-92 results in accelerated differentiation and increased triglyceride accumulation following hormonal stimulation, directly targeting the 3′ UTR region of Rb2/p130 (known to be involved in cell cycle regulation), which explains the subsequent reduction in Rb2/p130 mRNA and protein levels during the clonal expansion phase. These data indicate that miR-17-92 promotes adipocyte differentiation by targeting and downregulating Rb2/p130. Consequently, miR-17-92 shifts the balance from proliferation to differentiation [78]. The same is observed for miR-125b-5p, which impairs G1/S phase transition as well as the mRNA and protein expression of G1/S-related genes, such as Cyclin D2, Cyclin D_3_, and CDK4 in 3T3-L1 preadipocytes [79]. Adipose tissue is known to exert some of its systemic effects through the storage and release of lipids, the secretion of adipokines, and by serving as a site of chronic low-grade inflammation in obesity [80,81]. Thus, interventions that preserve miRNA processing in adipose tissue may represent a potential approach for reducing obesity-related complications, inflammation, and associated diseases such as diabetes [58].

Studies have reported unique sets of miRNAs when comparing gene expression pro files between preadipocytes and mature adipocytes and between adipose tissue from lean and obese individuals, or in the presence or absence of inflammatory stimulus, or between abdominal and subcutaneous white adipose tissue (WAT) [47,82,83]. These findings suggest that low-grade chronic inflammation in obese individuals may regulate miRNA expression and thereby alter adipocyte function [51,84,85,86]. Martinelli et al. found that miR-519d, a miRNA whose expression is upregulated during adipocyte differentiation, was overexpressed in the SAT of obese individuals compared to the SAT of lean individuals. MiR-519d binds to the 3′ UTR region of *PPARA*, decreasing its expression. This effect may be associated with metabolic imbalance and the subsequent adipocyte hypertrophy in the subcutaneous adipose tissue of severely obese individuals [83] (See Table 1).

More importantly, it has been demonstrated that miRNAs that modulate adipogenesis [46,47] are expressed in human abdominal and subcutaneous WAT [82] and circulate in the blood as stable compounds capable of regulating other targets [87,88].

Multiple epigenetic effects of calcitriol have been described in various pathophysiological contexts and in different experimental models [30,89], including during the regulation of miRNAs involved in various processes related to adipocytes, both in vitro and in vivo [54,85,90,91]. Additionally, a correlation exists between low serum levels of calcitriol and increased Body Mass Index (BMI) and body fat [84]. Thus, calcitriol, traditionally known for its role in calcium homeostasis and bone health, has emerged as a potential modulator of adipose tissue function [84,92,93]. In fact, adipocytes express enzymes involved in vitamin D_3_ metabolism, store vitamin D_3_ in their droplets, and metabolize vitamin D_3_, which may mediate anti-inflammatory effects in adipose tissue [84,94,95,96].

Studies suggest that calcitriol exerts catabolic effects on adipocytes, reducing lipid accumulation [30,89]. Analysis of the vitamin D_3_ receptor (VDR) protein revealed that its expression is higher in undifferentiated 3T3-L1 and hAMSC cells and during the early stages of adipogenesis, but that its expression subsequently decreases in both cell models. The calcitriol-dependent epigenetic mechanism that underpins the regulation of adipogenesis in3T3-L1 cells and hAMSC cells involves the upregulation of miR-27a-3p and miR-27b-3p [30]. In fact, previous studies have demonstrated that miR-27a-3p and miR-27b-3p have antiadipogenic effects and can regulate PPARγ and C/EBPα expression in both mouse adipose cell models, and in human models [59]. Additionally, Xuejun Ge et al. demonstrated that the promoter region of miR-27a-3p contains three putative VDR binding sites and one VDR binding site in the miR-27b-3p promoter, through which calcitriol increases the 321 expression of miR-27a-3p and miR-27b-3p transcripts in human oral keratinocytes [97]. These findings demonstrate a novel epigenetic modulation of adipocyte 323 differentiation by calcitriol.

Adipose tissue inflammation is believed to be a contributing factor to many chronic diseases that are associated with obesity. This metabolic inflammation is also characterized by increased miRNA production [43,50,91]. Calcitriol is known to limit this metabolic inflammation by decreasing the expression of inflammatory markers and leukocyte infiltration into adipose tissue [98], as well as decreasing miRNA expression in adipose tissue in mice and humans [91]. Vitamin D deficiency, defined by plasma levels of 25-hydroxyvitamin D_3_ (a recognized clinical marker) of below 50 nmol/L, is reported to demonstrate a negative correlation with inflammatory markers [99].

TNFα-mediated inflammation increases the levels of both inflammatory miR-146a and miR-155 in mature adipocytes, and calcitriol could attenuate this effect [88]. In human adipocytes, pre-incubation with 1,25-D_3_ prevented the TNFα-induced increase in miR-146a, miR-150, and miR-155. These results were also observed in vivo studies where animals were fed with a high-fat diet and vitamin D_3_ supplementation successfully inhibited the increase in these miRNAs [91]. Calcitriol’s ability to inactivate NF-κB signaling, by inhibition of p65 and IκB phosphorylation in murine adipocytes, could constitute a key molecular mechanism and, thus, represent a new mechanism by which calcitriol regulates inflammation [91]. In fact, bioinformatic analysis has indicated that genes regulated by calcitriol and miRNAs converge on the canonical nuclear factor kappa B (NF-κB) signaling pathway [54].

It has also recently been demonstrated that biological fluids such as plasma, serum, semen, urine, and saliva contain a significant number of extracellular miRNAs [100,101]. These miRNAs could be incorporated into extracellular vesicles (EVs) to modulate the activity of target cells such as those in brain, pancreas, and liver [50,102,103,104]. The analysis of the miRNA content of isolated EVs derived from mature human adipocytes revealed that TNFα-mediated inflammation significantly increased the expression of miR-155 and that calcitriol could dampen its effect, reducing inflammation in target cells [88]. MicroRNAs can also modulate VDR expression in adipose tissue via EVs. MiR-122-enriched exosome like vesicles derived from fat tissue could promote adipogenic differentiation by targeting VDR to modulate *SREBF1* during adipogenesis [105].

Calcitriol plays a regulatory role in adipocyte differentiation, adipose tissue energy metabolism, and inflammation, thereby acting as a modulator in adipose tissue. Calcitriol and miRNAs interact synergistically to regulate gene expression, influencing not only local adipose tissue metabolism, but also target organs via adipose-secreted factors. Consequently, these miRNAs represent promising therapeutic targets for the treatment of metabolic dysfunctions associated with obesity and its related diseases, as summarized in Table 2.

#### 1.3.2. Interplay Between 1α,25(OH)_2_ Vitamin D_3_ and the AMPK-SIRT1 Axis in Cellular Often Demonstrate Modest or No Effects on Meta Energy Homeostasis and Metabolic Regulation

AMP-activated protein kinase (AMPK) and sirtuin 1 (SIRT1) are central sensors of cellular energy status. AMPK is activated under conditions of energy stress, promoting increased glucose uptake, lipid oxidation, and mitochondrial biogenesis. In contrast, SIRT1, an NAD-dependent deacetylase, regulates processes such as metabolism, inflammation, and cellular aging. Recent studies indicate that these pathways act in an integrated manner, forming a functional axis that coordinates cellular metabolic adaptation [106,107].

In this scenario, calcitriol has been described as a modulator of these energy pathways. Evidence suggests that the active form of vitamin D_3_, calcitriol, can induce AMPK activation and increase SIRT1 expression, promoting beneficial metabolic effects, including improved insulin sensitivity and reduced oxidative stress [108,109]. Furthermore, AMPK activation can increase NAD availability, favoring SIRT1 activation, which reinforces the existence of a positive feedback loop modulated by calcitriol.

The crosstalk between calcitriol and the AMPK–SIRT1 axis represents one of the main emerging mechanisms in the regulation of energy metabolism. Recent studies demonstrate that calcitriol can act through both genomic mechanisms (classical pathway), via VDR, and non-genomic (non-classical pathways), directly influencing intracellular signaling cascades. This integrated effect allows calcitriol to modulate processes such as fatty acid oxidation, glucose metabolism, and metabolic inflammation [110,111].

Additionally, the interaction between calcitriol and SIRT1 has been associated with the regulation of inflammation and oxidative stress, factors closely linked to metabolic dysfunction. Studies indicate that calcitriol can increase SIRT1 activity, contributing to the deacetylation of important targets such as PGC-1α, thereby promoting metabolic adaptation and improving mitochondrial function [112,113].

**Figure 1:**
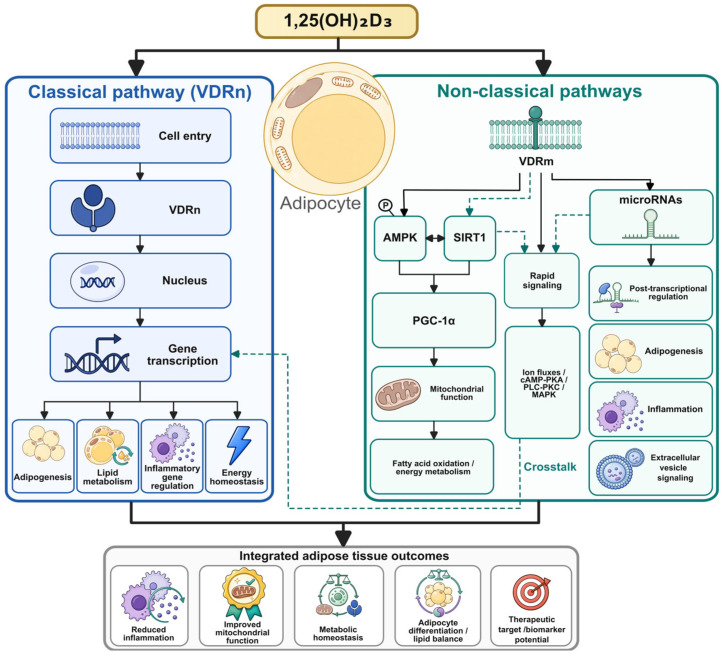
Integrative model of active vitamin D_3_ [1α,25-D_3_] signaling in adipose tissue. The classical pathway (blue) relies on the cellular entry of 1α,25-D_3_ and binding to the nuclear vitamin D_3_ receptor (VDRn). This binding promotes transcriptional regulation of genes associated with adipogenesis, lipid metabolism, inflammation, and energy homeostasis. In contrast, the non-classical pathways (green) utilize membrane-associated vitamin D_3_ receptors (VDRm) to activate proteins such as AMPK, SIRT1, and PGC-1α, thereby modulating mitochondrial function, fatty acid oxidation, energy metabolism, and microRNA-mediated post-transcriptional regulation. The crosstalk between these pathways reduces inflammation, improves mitochondrial function, and modulates metabolic homeostasis and adipocyte differentiation, highlighting the therapeutic and biomarker potential of 1α,25-D_3_ signaling in adipose tissue.

Energy metabolism, thus, emerges as the central axis of the non-classical effects of calcitriol. Activation of the AMPK–SIRT1 axis converges on the regulation of the coactivator PGC-1α, considered the main regulator of mitochondrial biogenesis. This process results in increased cellular oxidative capacity, improved mitochondrial efficiency, and reduced production of reactive oxygen species, contributing to energy homeostasis [114,115]. More recently, evidence demonstrates that calcitriol can directly improve mitochondrial function by regulating oxygen consumption and mitochondrial dynamics, as well as promoting metabolic flexibility. These effects are particularly relevant in pathological conditions such as obesity, type 2 diabetes, and metabolic syndrome, in which cellular bioenergetics is impaired [116,117]. The interplay among different pathways of calcitriol in adipocytes is depicted in Figure 1.

## 2. Methods

The present study consists of a narrative review of scientific literature, developed with the aim of gathering, analyzing, and discussing current evidence on both classical and non-classical mechanisms of action of calcitriol, with particular emphasis on its role in the regulation of energy metabolism and adipose tissue biology. This review adopts an integrative perspective, addressing the complexity of molecular, metabolic, and epigenetic networks involved in the action of calcitriol across different physiological and pathological contexts.

The literature search was conducted using the PubMed, ScienceDirect, and MDPI indexed journals databases, which are widely recognized for their comprehensive coverage in the biomedical field. For the PubMed database, studies published within the last 10 years were prioritized to ensure up-to-date scientific evidence. The following descriptors were used, either individually or in combination: 1,25-D_3_, calcitriol, vitamin D receptor, adipose tissue, adipocyte, energy metabolism, inflammation, insulin sensitivity, microRNAs, AMPK, SIRT1, mitochondrial function, lipogenesis, lipolysis, and metabolic diseases.

Study selection was based on relevance to the proposed topic, methodological quality and contribution to the understanding of biological mechanisms associated with calcitriol in the context of energy metabolism. Review articles, systematic reviews, meta-analyses, randomized clinical trials, experimental studies, and high-quality observational studies were prioritized. Mechanistic studies, including both in vitro and in vivo models, were also included when they provided relevant evidence on molecular pathways, particularly those related to metabolic, inflammatory, and mitochondrial regulation.

As this work was designed as a narrative review, no formal risk of bias assessment or quantitative synthesis was performed. The selected studies were analyzed through a critical and interpretative approach, considering the consistency of findings, the biological plausibility of the proposed mechanisms, and relevance to building an integrated understanding of the role of calcitriol in the regulation of energy metabolism.

## 3. Methodological Limitations and Translational Challenges

The interpretation of the reported effects of calcitriol on energy metabolism is limited by important methodological issues. In vitro studies often use calcitriol concentrations that exceed physiological levels, potentially overestimating biological effects [13]. Furthermore, cell lines such as 3T3-L1 do not fully reproduce human adipose tissue heterogeneity, particularly with regard to the inflammatory microenvironment and depot-specific differences [8].

In animal studies, differences in physiology, metabolism, and adipose tissue distribution limit direct extrapolation to humans. In clinical studies, lack of standardization in assessing calcitriol status and interindividual variability in response to supplementation hinder definitive conclusions [118,119,120]. Another relevant aspect is the possible existence of a “calcitriol resistance” state in individuals with obesity, characterized by alterations in VDR expression and function, chronic inflammation, and changes in the cellular microenvironment, which may impair metabolic responses to calcitriol [16,22].

Experimental models, including cell cultures and animal studies, provide relatively consistent evidence that calcitriol may positively influence energy metabolism. In rodents, calcitriol supplementation has been associated with improved insulin sensitivity, reduced inflammation, and modulation of lipid metabolism [13,121]. However, human studies report more heterogeneous results. Randomized clinical trials evaluating calcitriol supplementation in individuals with obesity or type 2 diabetes often demonstrate modest no effects on metabolic parameters, such as lipid profile, insulin resistance, and body composition [119,120,122].

This discrepancy may be explained by factors such as differences in the dose and duration of supplementation, variability in baseline calcitriol levels, heterogeneity of study populations, and the influence of confounding factors such as diet, physical activity, and body composition [118]. Additionally, the complexity of human metabolism and the presence of compensatory mechanisms may attenuate effects observed in experimental models. In addition, the greatest barrier to understanding the dose-response or concentration response of calcitriol in whole organisms (human or experimental models) or in isolated cells (primary culture or cell line) is the enzymatic machinery of vitamin D_3_ that is present in the organs. The enzymes responsible for vitamin D_3_ activation and degradation can dynamically regulate tissue and circulating calcitriol levels, thereby obscuring direct relationships between administered doses and biological responses.

## 4. Pathophysiological Implications and Future Directions

Although experimental evidence suggests that calcitriol plays a relevant role in the regulation of lipid and energy metabolism, findings in humans remain inconclusive. This discrepancy highlights the need for better-controlled studies that consider adipose tissue heterogeneity, metabolic status, and the complexity of calcitriol signaling.

Vitamin D_3_ deficiency has been associated with metabolic disorders, including insulin resistance, chronic low-grade inflammation, and mitochondrial dysfunction. These effects appear to be related, at least in part, to dysregulation of the AMPK–SIRT1 axis, reinforcing the role of calcitriol as a modulator of energy homeostasis [28,29]. Conversely, maintaining adequate levels of calcitriol may promote beneficial metabolic reprogramming, increasing bioenergetic efficiency and reducing the risk of metabolic diseases.

A comprehensive understanding of the molecular mechanisms governing classical and non-classical calcitriol signaling in preadipocytes and adipocytes is essential for elucidating the therapeutic potential of calcitriol in metabolic diseases. In this regard, miRNAs have emerged as key regulatory molecules capable of fine-tuning intracellular signaling networks by enhancing or repressing gene expression and downstream pathways. Moreover, miRNAs can be selectively incorporated into extracellular vesicles and delivered to distant tissues, where they participate in intercellular communication and the maintenance of metabolic homeostasis. These properties position miRNAs as promising therapeutic targets and minimally invasive biomarkers, offering new opportunities for the diagnosis, prognosis, and treatment of metabolic disorders.

## 5. Conclusions

In conclusion, recent advances consolidate a new perspective on calcitriol, positioning it as a central regulator of energy metabolism. The integration between classical calcitriol VDRn signaling, the AMPK–SIRT1–PGC-1α axis and miRNAs significantly expands our understanding of its biological effects, where mapping these pathways provides context for the precise and coordinated development of novel therapeutics for metabolic diseases. Further mechanistic and clinical research is warranted to clarify the contexts and molecular pathways through which modulation of the VD/VDRn/AMPK-SIRT1-PGC-1α/miRNAs axis can be effectively harnessed for metabolic and adipose tissue regulation.

## Figures and Tables

**Table 1:** MicroRNAs Involved in Adipogenesis.

miRNA	Effect on Adipogenesis	Target	Cell	Reference
miR-27a and miR-27b	↓	PPARγ	3T3-L1, hMADS	[59,62,63,64]
miR-145	↓	IRS1, PI3K/Akt and MAPK signaling pathways, PPARγ, C/EBPα, *FABP4*	Porcine preadipocytes, 3T3-L1, bovine preadipocyte	[31,61,65]
miR-143	↑	ERK5	Human white preadipocytes	[68]
miR-26a and miR-26b	↑	ADAM17	hMADS	[44]
miR-30c	↑	PAI-1 and ALK2	hMADS	[69]
miR-148a	↑	Wnt1	hMADS	[72]
miR-183	↑	Wnt/β-catenin	3T3-L1	[73]
miR-17-92	↑	Rb2/p130	3T3-L1	[78]
miR-125b-5p	↑	Cyclin D2, Cyclin D3, and CDK4	3T3-L1	[79]
miR-519d	↑	PPARα	SAT	[83]

↓, inhibition; ↑, promotes; hMADS, human multipotent adipose-derived stem cells; SAT, subcutaneous adipose tissue.

**Table 2:** Calcitriol effects on microRNAs in Adipocytes.

MicroRNA	Effect (Cell)	Reference
↑ miR-27a-3p miR-27b-3p	↓ adipogenesis (3T3-L1 and hAMSC)	[30]
↓ miR-146a ↓ miR-155	↓ inflammation (SGBS)	[88]
↓ miR-146a ↓ miR-150 ↓ miR-155	↓ inflammation (human and mouse adipocytes, 3T3-L1)	[91]

↓, decrease; ↑, increase; hAMSCs, human amniotic mesenchymal stromal cells; Simpson–Golabi–Behmel syndrome (SGBS) preadipocytes.

## Data Availability

Not applicable.

## Abbreviations

The following abbreviations are used in this manuscript:

1,25-D_3_	1α,25(OH)_2_ Vitamin D_3_
3′UTR	3′ Untranslated Region
ACC1	Acetyl-CoA Carboxylase 1
ADAM17	A Disintegrin and Metalloproteinase 17
ALK-2	Activin Receptor-Like Kinase 2
AMPK	AMP-Activated Protein Kinase
aP2	Adipocyte Protein 2
ATGL	Adipose Triglyceride Lipase
BMI	Body Mass Index
C/EBP	CCAAT/Enhancer-Binding Protein
CDK4	Cyclin-Dependent Kinase 4
c-Myc	Cellular Myelocytomatosis
CREB	cAMP Response Element-Binding Protein
ERK5	Extracellular Signal-Regulated Kinase 5
EVs	Extracellular Vesicles
*FABP4*	Fatty Acid-Binding Protein 4
FAS	Fas Cell Surface Death Receptor
G1/S	Gap 1/Synthesis Phase
hAMSC	Human Amniotic Mesenchymal Stromal Cells
HIF-1α	Hypoxia-Inducible Factor 1 Alpha
hMADS	Human Multipotent Adipose-Derived Stem Cells
HSL	Hormone-Sensitive Lipase
IL-6	Interleukin 6
IRS1	Insulin Receptor Substrate 1
IκB	Inhibitor of Kappa B
Let-7	Lethal-7
lncRNAs	Long Non-Coding RNAs
*LPL*	Lipoprotein Lipase
MAPK	Mitogen-Activated Protein Kinase
MCP-1	Monocyte Chemoattractant Protein 1
miRNAs	MicroRNAs
mRNA	Messenger RNA
NAD^+^	Nicotinamide Adenine Dinucleotide
ncRNAs	Non-Coding RNAs
NF-κB	Nuclear Factor Kappa B
PAI-1	Plasminogen Activator Inhibitor 1
PGC-1α	Peroxisome Proliferator-Activated Receptor Gamma Coactivator 1 Alpha
PI3K/AKT	Phosphoinositide 3-Kinase/Protein Kinase B
PPAR-γ	Peroxisome Proliferator-Activated Receptor Gamma
Pref-1	Preadipocyte Factor 1
Rb2/p130	Retinoblastoma-Like Protein 2
SAT	Subcutaneous Adipose Tissue
*SCD*-1	Stearoyl-CoA Desaturase 1
SIRT1	Sirtuin 1
SREBP-1c	Sterol Regulatory Element-Binding Protein 1c
TNF-α	Tumor Necrosis Factor Alpha
VAT	Visceral Adipose Tissue
VDR	Vitamin D Receptor
VitD	Vitamin D
WAT	White Adipose Tissue
Wnt	Wingless/Integrated 1

## References

[1] Chew S.M., Liu B., Shen S., Iyengar N.M. (2024). The Role of Obesity and Inflammation in Breast Cancer Recurrence. Curr. Breast Cancer Rep..

[2] Dourado T.M., Tirapelli C.R. (2026). The Role of Perivascular Adipose Tissue, White Adipose Tissue, and Brown Adipose Tissue in the Pathophysiological Effects of Ethanol. Am. J. Pathol..

[3] Sehgal R., Haacke N., Maguolo A., Solari F.A., Jähnert M., Gottmann P., Nilsson E., Vaag A., Fischer-Posovszky P., Werberger A. (2025). Adipose tissue-derived microRNAs as epigenetic modulators of type 2 diabetes. BMC Med..

[4] Wang J., Zhang X., Zhu Y., Sun H., Chen X., Zhao Z., Zhang N., Zhang C., Li L., Bi Y. (2026). Adipocyte-derived extracellular vesicles are key regulators of central leptin sensitivity and energy homeostasis. Cell Metab..

[5] Harford K.A., Reynolds C.M., McGillicuddy F.C., Roche H.M. (2011). Fats, inflammation and insulin resistance: Insights to the role of macrophage and T-cell accumulation in adipose tissue. Proc. Nutr. Soc..

[6] Liang Z., Wang Z., Liu X., He Y. (2024). Confronting the global obesity epidemic: Investigating the role and underlying mechanisms of vitamin D in metabolic syndrome management. Front. Nutr..

[7] Ibrahim M.M. (2010). Subcutaneous and visceral adipose tissue: Structural and functional differences. Obes. Rev..

[8] Tchkonia T., Thomou T., Zhu Y., Karagiannides I., Pothoulakis C., Jensen M., Kirkland J. (2013). Mechanisms and Metabolic Implications of Regional Differences among Fat Depots. Cell Metab..

[9] Karastergiou K., Fried S.K., Xie H., Lee M.J., Divoux A., Rosencrantz M.A., Chang R.J., Smith S.R. (2013). Distinct Developmental Signatures of Human Abdominal and Gluteal Subcutaneous Adipose Tissue Depots. J. Clin. Endocrinol. Metab..

[10] Wajchenberg B.L. (2000). Subcutaneous and Visceral Adipose Tissue: Their Relation to the Metabolic Syndrome. Endocr. Rev..

[11] Gupta V.K., Sahu L., Sonwal S., Suneetha A., Kim D.H., Kim J., Verma H.K., Pavitra E., Raju G.S.R., Bhaskar L. (2024). Advances in biomedical applications of vitamin D for VDR targeted management of obesity and cancer. Biomed. Pharmacother..

[12] Jirků J., Kršáková Z., Křížová J. (2026). Vitamin D in Obesity: Mechanisms and Clinical Impact. Obesities.

[13] Ding C., Gao D., Wilding J., Trayhurn P., Bing C. (2012). Vitamin D signalling in adipose tissue. Br. J. Nutr..

[14] Park C.Y., Han S.N. (2021). The Role of Vitamin D in Adipose Tissue Biology: Adipocyte Differentiation, Energy Metabolism, and Inflammation. J. Lipid Atheroscler..

[15] Weisberg S.P., McCann D., Desai M., Rosenbaum M., Leibel R.L., Ferrante A.W. (2003). Obesity is associated with macrophage accumulation in adipose tissue. J. Clin. Investig..

[16] Lee M.J. (2025). Vitamin D Enhancement of Adipose Biology: Implications on Obesity-Associated Cardiometabolic Diseases. Nutrients.

[17] Trayhurn P. (2013). Hypoxia and Adipose Tissue Function and Dysfunction in Obesity. Physiol. Rev..

[18] Shantavasinkul P.C., Nimitphong H. (2022). Vitamin D and Visceral Obesity in Humans: What Should Clinicians Know?. Nutrients.

[19] Argan R.J.A., Alqatari S.G., Alwaheed A.J., Hasan M.A., AlQahtani S.Y., Shubbar M.D.A., Alnasser A.H., Abbas S.M.A., AlYousef N.H. (2026). Vitamin D deficiency in obesity: Epidemiological evidence, biological mechanisms, and clinical considerations. Obes. Med..

[20] Lay S.L., Scherer P.E. (2025). Exploring adipose tissue-derived extracellular vesicles in inter-organ crosstalk: Implications for metabolic regulation and adipose tissue function. Cell Rep..

[21] Blumberg J.M., Tzameli I., Astapova I., Lam F.S., Flier J.S., Hollenberg A.N. (2006). Complex Role of the Vitamin D Receptor and Its Ligand in Adipogenesis in 3T3-L1 Cells. J. Biol. Chem..

[22] Cimini F.A., Sentinelli F., Oldani A., Barchetta I., Cavallo M.G. (2025). Adipose Tissue Dysfunction and Metabolic Diseases: The Role of Vitamin D/Vitamin D Receptor Axis. Int. J. Mol. Sci..

[23] Marcotorchino J., Gouranton E., Romier B., Tourniaire F., Astier J., Malezet C., Amiot M., Landrier J. (2012). Vitamin D reduces the inflammatory response and restores glucose uptake in adipocytes. Mol. Nutr. Food Res..

[24] Wortsman J., Matsuoka L.Y., Chen T.C., Lu Z., Holick M.F. (2000). Decreased bioavailability of vitamin D in obesity. Am. J. Clin. Nutr..

[25] Norman A.W., Olivera C.J., Silva F.R.M.B., Bishop J.E. (2002). A specific binding protein/receptor for 1,25-dihydroxyvitamin D3 is present in an intestinal caveolae membrane fraction. Biochem. Biophys. Res. Commun..

[26] Norman A.W., Mizwicki M.T., Norman D.P.G. (2004). Steroid-hormone rapid actions, membrane receptors and a conformational ensemble model. Nat. Rev. Drug Discov..

[27] Sun X., Zemel M.B. (2004). Role of uncoupling protein 2 (UCP2) expression and 1, 25-dihydroxyvitamin D 3 in modulating adipocyte apoptosis. FASEB J..

[28] Bouillon R., Manousaki D., Rosen C., Trajanoska K., Rivadeneira F., Richards J.B. (2022). The health effects of vitamin D supplementation: Evidence from human studies. Nat. Rev. Endocrinol..

[29] Muscogiuri G. (2020). Introduction to Vitamin D: Current evidence and future directions. Eur. J. Clin. Nutr..

[30] Provvisiero D.P., Negri M., Amatrudo F., Patalano R., Montò T., de Angelis C., Graziadio C., Pugliese G., de Alteriis G., Colao A. (2024). 1,25-Dihydroxyvitamin D3 mitigates the adipogenesis induced by bisphenol A in 3T3-L1 and hAMSC through miR-27-3p regulation. Int. J. Obes..

[31] Wang L., Zhang S., Cheng G., Mei C., Li S., Zhang W., Junjvlieke Z., Zan L. (2020). MiR-145 reduces the activity of PI3K/Akt and MAPK signaling pathways and inhibits adipogenesis in bovine preadipocytes. Genomics.

[32] Pisani D.F., Ghandour R.A., Beranger G.E., Faouder P.L., Chambard J.C., Giroud M., Vegiopoulos A., Djedaini M., Bertrand-Michel J., Tauc M. (2014). The 6-fatty acid, arachidonic acid, regulates the conversion of white to brite adipocyte through a prostaglandin/calcium mediated pathway. Mol. Metab..

[33] Yore M., Syed I., Moraes-Vieira P., Zhang T., Herman M., Homan E., Patel R., Lee J., Chen S., Peroni O. (2014). Discovery of a Class of Endogenous Mammalian Lipids with Anti-Diabetic and Anti-inflammatory Effects. Cell.

[34] Wei S., Du M., Jiang Z., Hausman G.J., Zhang L., Dodson M.V. (2016). Long noncoding RNAs in regulating adipogenesis: New RNAs shed lights on obesity. Cell. Mol. Life Sci..

[35] Lee R.C., Feinbaum R.L., Ambros V. (1993). The C. elegans heterochronic gene lin-4 encodes small RNAs with antisense complementarity to lin-14. Cell.

[36] Wahid F., Shehzad A., Khan T., Kim Y.Y. (2010). MicroRNAs: Synthesis, mechanism, function, and recent clinical trials. Biochim. Biophys. Acta (BBA)—Mol. Cell Res..

[37] van Rooij E., Purcell A.L., Levin A.A. (2012). Developing MicroRNA Therapeutics. Circ. Res..

[38] Hydbring P., Badalian-Very G. (2013). Clinical applications of microRNAs. F1000Research.

[39] Hayes J., Peruzzi P.P., Lawler S. (2014). MicroRNAs in cancer: Biomarkers, functions and therapy. Trends Mol. Med..

[40] Wahid F., Khan T., Kim Y.Y. (2014). MicroRNA and diseases: Therapeutic potential as new generation of drugs. Biochimie.

[41] Lagos-Quintana M., Rauhut R., Lendeckel W., Tuschl T. (2001). Identification of Novel Genes Coding for Small Expressed RNAs. Science.

[42] Vienberg S., Geiger J., Madsen S., Dalgaard L.T. (2017). MicroRNAs in metabolism. Acta Physiol..

[43] Arner P., Kulyté A. (2015). MicroRNA regulatory networks in human adipose tissue and obesity. Nat. Rev. Endocrinol..

[44] Karbiener M., Pisani D.F., Frontini A., Oberreiter L.M., Lang E., Vegiopoulos A., Mössenböck K., Bernhardt G.A., Mayr T., Hildner F. (2013). MicroRNA-26 Family Is Required for Human Adipogenesis and Drives Characteristics of Brown Adipocytes. Stem Cells.

[45] Scheideler M. (2016). MicroRNAs in adipocyte formation and obesity. Best Pract. Res. Clin. Endocrinol. Metab..

[46] Son Y.H., Ka S., Kim A.Y., Kim J.B. (2014). Regulation of Adipocyte Differentiation via MicroRNAs. Endocrinol. Metab..

[47] Xie H., Sun L., Lodish H.F. (2009). Targeting microRNAs in obesity. Expert Opin. Ther. Targets.

[48] Krützfeldt J., Stoffel M. (2006). MicroRNAs: A new class of regulatory genes affecting metabolism. Cell Metab..

[49] Iacomino G., Siani A. (2017). Role of microRNAs in obesity and obesity-related diseases. Genes Nutr..

[50] Landrier J.F., Derghal A., Mounien L. (2019). MicroRNAs in Obesity and Related Metabolic Disorders. Cells.

[51] McGregor R.A., Choi M.S. (2011). microRNAs in the Regulation of Adipogenesis and Obesity. Curr. Mol. Med..

[52] Bond S.T., Calkin A.C., Drew B.G. (2022). Adipose-Derived Extracellular Vesicles: Systemic Messengers and Metabolic Regulators in Health and Disease. Front. Physiol..

[53] Gao X., Salomon C., Freeman D.J. (2017). Extracellular Vesicles from Adipose Tissue—A Potential Role in Obesity and Type 2 Diabetes?. Front. Endocrinol..

[54] Karkeni E., Payet T., Astier J., Sicard F., Mounien L., Landrier J.F. (2023). Proposal of a bioinformatics approach to predict molecular mechanisms involved in inflammatory response: Case of ATRA and 1,25(OH) 2 D in adipocytes. Epigenetics.

[55] Moseti D., Regassa A., Kim W.K. (2016). Molecular Regulation of Adipogenesis and Potential Anti-Adipogenic Bioactive Molecules. Int. J. Mol. Sci..

[56] Ntambi J.M., Young-Cheul K. (2000). Adipocyte Differentiation and Gene Expression. J. Nutr..

[57] Rangwala S.M., Lazar M.A. (2000). Transcriptional control of adipogenesis. Annu. Rev. Nutr..

[58] Amri E.Z., Scheideler M. (2017). Small non coding RNAs in adipocyte biology and obesity. Mol. Cell. Endocrinol..

[59] Karbiener M., Fischer C., Nowitsch S., Opriessnig P., Papak C., Ailhaud G., Dani C., Amri E.Z., Scheideler M. (2009). microRNA miR-27b impairs human adipocyte differentiation and targets PPAR. Biochem. Biophys. Res. Commun..

[60] Lee E.K., Lee M.J., Abdelmohsen K., Kim W., Kim M.M., Srikantan S., Martindale J.L., Hutchison E.R., Kim H.H., Marasa B.S. (2011). miR-130 Suppresses Adipogenesis by Inhibiting Peroxisome Proliferator-Activated Receptor Expression. Mol. Cell. Biol..

[61] Guo Y., Mo D., Zhang Y., Zhang Y., Cong P., Xiao S., He Z., Liu X., Chen Y. (2012). MicroRNAome Comparison between Intramuscular and Subcutaneous Vascular Stem Cell Adipogenesis. PLoS ONE.

[62] Kim S.Y., Kim A.Y., Lee H.W., Son Y.H., Lee G.Y., Lee J.W., Lee Y.S., Kim J.B. (2010). miR-27a is a negative regulator of adipocyte differentiation via suppressing PPARγ expression. Biochem. Biophys. Res. Commun..

[63] Zou B., Ge Z., Zhu W., Xu Z., Li C. (2015). Persimmon tannin represses 3T3-L1 preadipocyte differentiation via up-regulating expression of miR-27 and down-regulating expression of peroxisome proliferator-activated receptor- in the early phase of adipogenesis. Eur. J. Nutr..

[64] Gan C.C., Ni T.W., Yu Y., Qin N., Chen Y., Jin M.N., Duan H.Q. (2017). Flavonoid derivative (Fla-CN) inhibited adipocyte differentiation via activating AMPK and up-regulating microRNA-27 in 3T3-L1 cells. Eur. J. Pharmacol..

[65] Du J., Cheng X., Shen L., Tan Z., Luo J., Wu X., Liu C., Yang Q., Jiang Y., Tang G. (2016). Methylation of miR-145a-5p promoter mediates adipocytes differentiation. Biochem. Biophys. Res. Commun..

[66] Lorente-Cebrián S., Mejhert N., Kulyté A., Laurencikiene J., Åström G., Hedén P., Rydén M., Arner P. (2014). MicroRNAs Regulate Human Adipocyte Lipolysis: Effects of miR-145 Are Linked to TNF-α. PLoS ONE.

[67] Lin Y.Y., Chou C.F., Giovarelli M., Briata P., Gherzi R., Chen C.Y. (2014). KSRP and MicroRNA 145 Are Negative Regulators of Lipolysis in White Adipose Tissue. Mol. Cell. Biol..

[68] Esau C., Kang X., Peralta E., Hanson E., Marcusson E.G., Ravichandran L.V., Sun Y., Koo S., Perera R.J., Jain R. (2004). MicroRNA-143 Regulates Adipocyte Differentiation. J. Biol. Chem..

[69] Karbiener M., Neuhold C., Opriessnig P., Prokesch A., Bogner-Strauss J.G., Scheideler M. (2011). MicroRNA-30c promotes human adipocyte differentiation and co-repressesPAI-1andALK2. RNA Biol..

[70] Lim L.P., Lau N.C., Garrett-Engele P., Grimson A., Schelter J.M., Castle J., Bartel D.P., Linsley P.S., Johnson J.M. (2005). Microarray analysis shows that some microRNAs downregulate large numbers of target mRNAs. Nature.

[71] Baek D., Villén J., Shin C., Camargo F.D., Gygi S.P., Bartel D.P. (2008). The impact of microRNAs on protein output. Nature.

[72] Shi C., Zhang M., Tong M., Yang L., Pang L., Chen L., Xu G., Chi X., Hong Q., Ni Y. (2015). miR-148a is Associated with Obesity and Modulates Adipocyte Differentiation of Mesenchymal Stem Cells through Wnt Signaling. Sci. Rep..

[73] Chen C., Xiang H., Peng Y.-L., Peng J., Jiang S.-W. (2014). Mature miR-183, negatively regulated by transcription factor GATA3, promotes 3T3-L1 adipogenesis through inhibition of the canonical Wnt/-catenin signaling pathway by targeting LRP6. Cell. Signal..

[74] Hamam D., Ali D., Kassem M., Aldahmash A., Alajez N.M. (2015). microRNAs as Regulators of Adipogenic Differentiation of Mesenchymal Stem Cells. Stem Cells Dev..

[75] Patel Y.M., Lane M. (2000). Mitotic Clonal Expansion during Preadipocyte Differentiation: Calpain-mediated Turnover of p27. J. Biol. Chem..

[76] Hayashita Y., Osada H., Tatematsu Y., Yamada H., Yanagisawa K., Tomida S., Yatabe Y., Kawahara K., Sekido Y., Takahashi T. (2005). A Polycistronic MicroRNA Cluster, miR-17-92, Is Overexpressed in Human Lung Cancers and Enhances Cell Proliferation. Cancer Res..

[77] Olive V., Jiang I., He L. (2010). mir-17-92, a cluster of miRNAs in the midst of the cancer network. Int. J. Biochem. Cell Biol..

[78] Wang Q., Li Y.C., Wang J., Kong J., Qi Y., Quigg R.J., Li X. (2008). miR-17-92 cluster accelerates adipocyte differentiation by negatively regulating tumor-suppressor Rb2/p130. Proc. Natl. Acad. Sci. USA.

[79] Ouyang D., Ye Y., Guo D., Yu X., Chen J., Qi J., Tan X., Zhang Y., Ma Y., Li Y. (2015). MicroRNA-125b-5p inhibits proliferation and promotes adipogenic differentiation in 3T3-L1 preadipocytes. Acta Biochim. Biophys. Sin..

[80] Kirichenko T.V., Markina Y.V., Bogatyreva A.I., Tolstik T.V., Varaeva Y.R., Starodubova A.V. (2022). The Role of Adipokines in Inflammatory Mechanisms of Obesity. Int. J. Mol. Sci..

[81] Ouchi N., Parker J.L., Lugus J.J., Walsh K. (2011). Adipokines in inflammation and metabolic disease. Nat. Rev. Immunol..

[82] Klöting N., Berthold S., Kovacs P., Schön M.R., Fasshauer M., Ruschke K., Stumvoll M., Blüher M. (2009). MicroRNA Expression in Human Omental and Subcutaneous Adipose Tissue. PLoS ONE.

[83] Martinelli R., Nardelli C., Pilone V., Buonomo T., Liguori R., Castanò I., Buono P., Masone S., Persico G., Forestieri P. (2010). miR-519d Overexpression Is Associated With Human Obesity. Obesity.

[84] Bennour I., Haroun N., Sicard F., Mounien L., Landrier J.F. (2022). Vitamin D and Obesity/Adiposity—A Brief Overview of Recent Studies. Nutrients.

[85] Jonas M.I., Kuryłowicz A., Bartoszewicz Z., Lisik W., Jonas M., Kozniewski K., Puzianowska-Kuznicka M. (2019). Vitamin D Receptor Gene Expression in Adipose Tissue of Obese Individuals is Regulated by miRNA and Correlates with the Pro-Inflammatory Cytokine Level. Int. J. Mol. Sci..

[86] Sultan S., Maashi M. (2025). Obesity Alters the microRNA Expression Profile Related to Metabolic Disorders in Peripheral Blood Mononuclear Cells: Preliminary Results. Curr. Issues Mol. Biol..

[87] Gilad S., Meiri E., Yogev Y., Benjamin S., Lebanony D., Yerushalmi N., Benjamin H., Kushnir M., Cholakh H., Melamed N. (2008). Serum MicroRNAs Are Promising Novel Biomarkers. PLoS ONE.

[88] Payet T., Astier J., Bournot L., Sicard F., Robert S., Lacroix R., Wabitsch M., Landrier J., Mounien L. (2024). Vitamin D modulates the content of inflammatory microRNAs in extracellular vesicles from human adipocyte cells in inflammatory context. BioFactors.

[89] Bouillon R., Carmeliet G., Lieben L., Watanabe M., Perino A., Auwerx J., Schoonjans K., Verstuyf A. (2013). Vitamin D and energy homeostasis—Of mice and men. Nat. Rev. Endocrinol..

[90] Cruciani S., Garroni G., Balzano F., Pala R., Bellu E., Cossu M.L., Ginesu G.C., Ventura C., Maioli M. (2020). Tuning Adipogenic Differentiation in ADSCs by Metformin and Vitamin D: Involvement of miRNAs. Int. J. Mol. Sci..

[91] Karkeni E., Bonnet L., Marcotorchino J., Tourniaire F., Astier J., Ye J., Landrier J.F. (2018). Vitamin D limits inflammation-linked microRNA expression in adipocytes in vitro and in vivo: A new mechanism for the regulation of inflammation by vitamin D. Epigenetics.

[92] Landrier J.F., Karkeni E., Marcotorchino J., Bonnet L., Tourniaire F. (2016). Vitamin D modulates adipose tissue biology: Possible consequences for obesity?. Proc. Nutr. Soc..

[93] Savastano S., Barrea L., Savanelli M.C., Nappi F., Somma C.D., Orio F., Colao A. (2017). Low vitamin D status and obesity: Role of nutritionist. Rev. Endocr. Metab. Disord..

[94] Bonnet L., Karkeni E., Couturier C., Astier J., Dalifard J., Defoort C., Svilar L., Martin J.C., Tourniaire F., Landrier J.F. (2017). Gene Expression Pattern in Response to Cholecalciferol Supplementation Highlights Cubilin as a Major Protein of 25(OH)D Uptake in Adipocytes and Male Mice White Adipose Tissue. Endocrinology.

[95] Bonnet L., Karkeni E., Couturier C., Astier J., Defoort C., Svilar L., Tourniaire F., Mounien L., Landrier J.F. (2021). Four days high fat diet modulates vitamin D metabolite levels and enzymes in mice. J. Endocrinol..

[96] Lontchi-Yimagou E., Kang S., Goyal A., Zhang K., You J.Y., Carey M., Jain S., Bhansali S., Kehlenbrink S., Guo P. (2020). Insulin-sensitizing effects of vitamin D repletion mediated by adipocyte vitamin D receptor: Studies in humans and mice. Mol. Metab..

[97] Ge X., Yuan L., Wei J., Nguyen T., Tang C., Liao W., Li R., Yang F., Zhang F., Zhao B. (2020). Vitamin D/VDR signaling induces miR-27a/b expression in oral lichen planus. Sci. Rep..

[98] Bournot L., Payet T., Sicard F., Breniere T., Astier J., Roux J., Bariohay B., Landrier J.F. (2024). Aging alone or combined with obesity increases white adipose tissue inflammatory status in male mice. Sci. Rep..

[99] Cannell J.J., Grant W.B., Holick M.F. (2014). Vitamin D and inflammation. Dermato-Endocrinology.

[100] Bei Y., Das S., Rodosthenous R.S., Holvoet P., Vanhaverbeke M., Monteiro M.C., Monteiro V.V.S., Radosinska J., Bartekova M., Jansen F. (2017). Extracellular Vesicles in Cardiovascular Theranostics. Theranostics.

[101] Schwarzenbach H., Nishida N., Calin G.A., Pantel K. (2014). Clinical relevance of circulating cell-free microRNAs in cancer. Nat. Rev. Clin. Oncol..

[102] Derghal A., Djelloul M., Trouslard J., Mounien L. (2017). The Role of MicroRNA in the Modulation of the Melanocortinergic System. Front. Neurosci..

[103] Guay C., Regazzi R. (2017). Exosomes as new players in metabolic organ cross-talk. Diabetes Obes. Metab..

[104] Thomou T., Mori M.A., Dreyfuss J.M., Konishi M., Sakaguchi M., Wolfrum C., Rao T.N., Winnay J.N., Garcia-Martin R., Grinspoon S.K. (2017). Adipose-derived circulating miRNAs regulate gene expression in other tissues. Nature.

[105] Huang X., Chen J., Ren Y., Fan L., Xiang W., He X. (2022). Exosomal miR-122 promotes adipogenesis and aggravates obesity through the VDR/SREBF1 axis. Obesity.

[106] Herzig S., Shaw R.J. (2018). AMPK: Guardian of metabolism and mitochondrial homeostasis. Nat. Rev. Mol. Cell Biol..

[107] Hajhashemy Z., Shahdadian F., Ziaei R., Saneei P. (2021). Serum vitamin D levels in relation to abdominal obesity: A systematic review and dose–response meta-analysis of epidemiologic studies. Obes. Rev..

[108] Zhu A., Zeng Y., Ji J.S. (2020). Residential Greenness Alters Serum 25(OH)D Concentrations: A Longitudinal Cohort of Chinese Older Adults. J. Am. Med. Dir. Assoc..

[109] Li A., Shen P., Liu S., Wang J., Zeng J., Du C. (2022). Vitamin D alleviates skeletal muscle loss and insulin resistance by inducing vitamin D receptor expression and regulating the AMPK/SIRT1 signaling pathway in mice. Food Sci. Technol..

[110] Barrea L., Frias-Toral E., Pugliese G., Garcia-Velasquez E., Carignano M.D.L.A., Savastano S., Colao A., Muscogiuri G. (2021). Vitamin D in obesity and obesity-related diseases: An overview. Minerva Endocrinol..

[111] Berridge M.J. (2015). Vitamin D cell signalling in health and disease. Biochem. Biophys. Res. Commun..

[112] Yang J., Zhang Y., Pan Y., Sun C., Liu Z., Liu N., Fu Y., Li X., Li Y., Kong J. (2021). The Protective Effect of 1,25(OH)_2_D_3_ on Myocardial Function is Mediated via Sirtuin 3-Regulated Fatty Acid Metabolism. Front. Cell Dev. Biol..

[113] Zhou Y., Zhang F., Ding J. (2022). As a Modulator, Multitasking Roles of SIRT1 in Respiratory Diseases. Immune Netw..

[114] Nemeth Z., Patonai A., Simon-Szabó L., Takács I. (2023). Interplay of Vitamin D and SIRT1 in Tissue-Specific Metabolism—Potential Roles in Prevention and Treatment of Non-Communicable Diseases Including Cancer. Int. J. Mol. Sci..

[115] Li Y., Kang K., Bao H., Liu S., Zhao B., Hu G., Wu J. (2025). Research Progress on the Interaction Between SIRT1 and Mitochondrial Biochemistry in the Aging of the Reproductive System. Biology.

[116] Wimalawansa S.J. (2019). Vitamin D Deficiency: Effects on Oxidative Stress, Epigenetics, Gene Regulation, and Aging. Biology.

[117] Szymczak-Pajor I., Miazek K., Selmi A., Balcerczyk A., Śliwińska A. (2022). The Action of Vitamin D in Adipose Tissue: Is There the Link between Vitamin D Deficiency and Adipose Tissue-Related Metabolic Disorders?. Int. J. Mol. Sci..

[118] Pilz S., Zittermann A., Trummer C., Theiler-Schwetz V., Lerchbaum E., Keppel M.H., Grübler M.R., März W., Pandis M. (2019). Vitamin D testing and treatment: A narrative review of current evidence. Endocr. Connect..

[119] Barbarawi M., Zayed Y., Barbarawi O., Bala A., Alabdouh A., Gakhal I., Rizk F., Alkasasbeh M., Bachuwa G., Manson J.E. (2020). Effect of Vitamin D Supplementation on the Incidence of Diabetes Mellitus. J. Clin. Endocrinol. Metab..

[120] Mendes A.K.B., Schlindwein H.W., Cabral L.W., Borba B.G.M., Silva F.R.M.B. (2022). Envolvimento do Metabólito Ativo da Vitamina D, 1,25(OH)_2_ Vitamina D3 Na Diabetes: Metabolismo De Glicose E Lipídios.

[121] Marcotorchino J., Tourniaire F., Astier J., Karkeni E., Canault M., Amiot M.J., Bendahan D., Bernard M., Martin J.C., Giannesini B. (2014). Vitamin D protects against diet-induced obesity by enhancing fatty acid oxidation. J. Nutr. Biochem..

[122] Autier P., Boniol M., Pizot C., Mullie P. (2014). Vitamin D status and ill health: A systematic review. Lancet Diabetes Endocrinol..

